# Supplementary material for: Genetic and Phenotypic Characterization of Multidrug-Resistant Klebsiella pneumoniae from Liver Abscess
Source: Microbiol Spectr. 2023 Jan 4;11(1):e02240-22. doi: 10.1128/spectrum.02240-22 (PMC9927449; doi:10.1128/spectrum.02240-22)
Supplement: Supplemental file 1 — Figures S1 and S2. Download spectrum.02240-22-s0001.pdf, PDF file, 1.35 MB [file spectrum.02240-22-s0001.pdf]

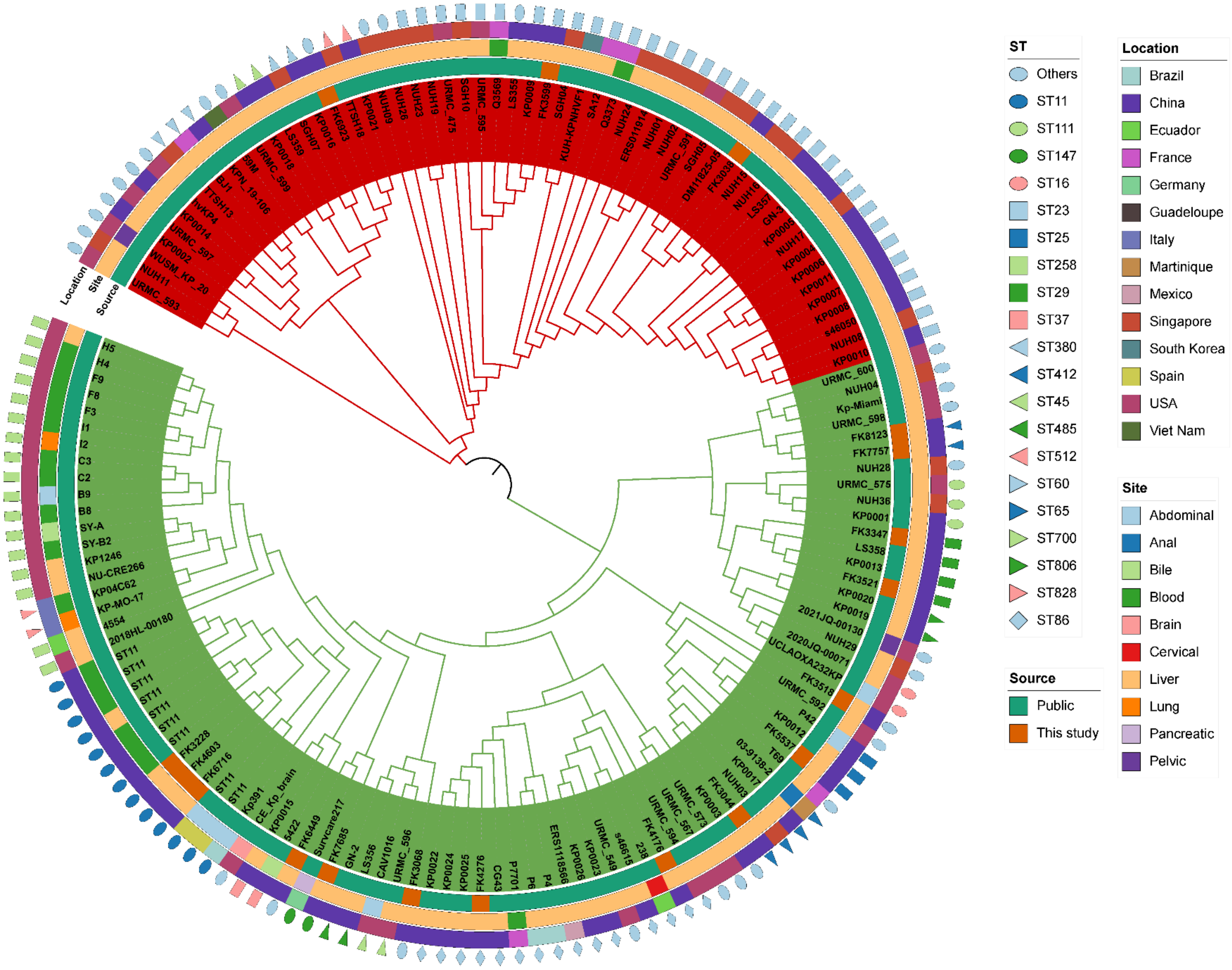

Figure S1. Phylogenetic tree of 112 LAKp with 36 *K. pneumoniae* isolated from various sites of patients from GenBank. Circles 1-5 (from outside to inside) represent the information as follows: (1) sequence type, (2) isolate location, (3) isolated site of patient, (4) source of sequences, (5) cluster of genomes (green indicates cluster 1, red indicates cluster 2).

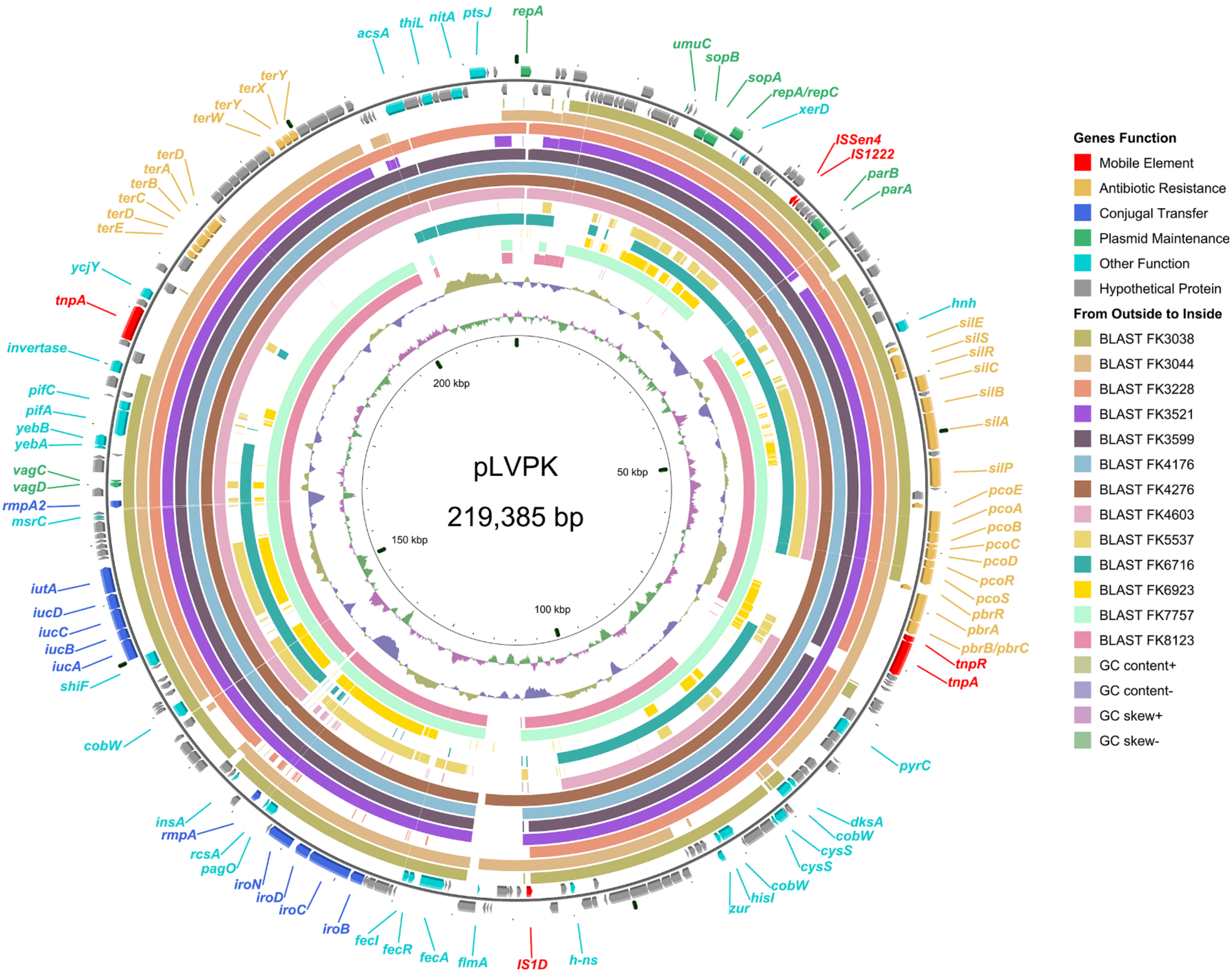

Figure S2. Alignment of assemblies of 13 pLVPK-positive genomes from MDR LAKP strains to the reference pLVPK plasmid (GenBank accession number AY378100). Functional features of pLVPK plasmid are highlighted in different colors.
